# Impact of Intrapartum Azithromycin on the Carriage and Antibiotic Resistance of *Escherichia coli* and *Klebsiella pneumoniae* in Mothers and Their Newborns: A Substudy of a Randomized, Double-Blind Trial Conducted in The Gambia and Burkina Faso

**DOI:** 10.1093/cid/ciae280

**Published:** 2024-05-16

**Authors:** Pauline Getanda, Isatou Jagne, Joel D Bognini, Bully Camara, Bakary Sanyang, Saffiatou Darboe, Ellen Sambou, Momodou Barry, Kady Kassibo, Aminata Cham, Harriet Mendy, Bintou K J Singateh, Ebrahim Ndure, Toussaint Rouamba, Abdoulie Bojang, Christian Bottomley, Benjamin P Howden, Umberto D’Alessandro, Halidou Tinto, Anna Roca, PregnAnZI-2 Carriage Study Group, Fatoumata Sillah, Nathalie Beloum, Usman N Nakakana, Madikoi Danso, Joquina C Jones, Shashu Graves, Edrissa Sabally, Siaka Badjie, Sulayman Bah, Omar B Jarra, Abdoulie Suso

**Affiliations:** Disease Control and Elimination Theme, Medical Research Council Unit, The Gambia at London School of Hygiene and Tropical Medicine (MRCG @ LSHTM), Banjul, The Gambia; Disease Control and Elimination Theme, Medical Research Council Unit, The Gambia at London School of Hygiene and Tropical Medicine (MRCG @ LSHTM), Banjul, The Gambia; Clinical Research Unit of Nanoro, Institut de Recherche en Sciences de la Santé, Nanoro, Burkina Faso; Disease Control and Elimination Theme, Medical Research Council Unit, The Gambia at London School of Hygiene and Tropical Medicine (MRCG @ LSHTM), Banjul, The Gambia; Disease Control and Elimination Theme, Medical Research Council Unit, The Gambia at London School of Hygiene and Tropical Medicine (MRCG @ LSHTM), Banjul, The Gambia; Disease Control and Elimination Theme, Medical Research Council Unit, The Gambia at London School of Hygiene and Tropical Medicine (MRCG @ LSHTM), Banjul, The Gambia; Disease Control and Elimination Theme, Medical Research Council Unit, The Gambia at London School of Hygiene and Tropical Medicine (MRCG @ LSHTM), Banjul, The Gambia; Disease Control and Elimination Theme, Medical Research Council Unit, The Gambia at London School of Hygiene and Tropical Medicine (MRCG @ LSHTM), Banjul, The Gambia; Disease Control and Elimination Theme, Medical Research Council Unit, The Gambia at London School of Hygiene and Tropical Medicine (MRCG @ LSHTM), Banjul, The Gambia; Disease Control and Elimination Theme, Medical Research Council Unit, The Gambia at London School of Hygiene and Tropical Medicine (MRCG @ LSHTM), Banjul, The Gambia; Disease Control and Elimination Theme, Medical Research Council Unit, The Gambia at London School of Hygiene and Tropical Medicine (MRCG @ LSHTM), Banjul, The Gambia; Disease Control and Elimination Theme, Medical Research Council Unit, The Gambia at London School of Hygiene and Tropical Medicine (MRCG @ LSHTM), Banjul, The Gambia; Disease Control and Elimination Theme, Medical Research Council Unit, The Gambia at London School of Hygiene and Tropical Medicine (MRCG @ LSHTM), Banjul, The Gambia; Clinical Research Unit of Nanoro, Institut de Recherche en Sciences de la Santé, Nanoro, Burkina Faso; Disease Control and Elimination Theme, Medical Research Council Unit, The Gambia at London School of Hygiene and Tropical Medicine (MRCG @ LSHTM), Banjul, The Gambia; Faculty of Infectious and Tropical Diseases, London School of Hygiene and Tropical Medicine, London, United Kingdom; Microbiological Diagnostic Unit Public Health Laboratory, Department of Microbiology and Immunology at the Peter Doherty Institute for Infection and Immunity, University of Melbourne, Melbourne, Victoria, Australia; Disease Control and Elimination Theme, Medical Research Council Unit, The Gambia at London School of Hygiene and Tropical Medicine (MRCG @ LSHTM), Banjul, The Gambia; Clinical Research Unit of Nanoro, Institut de Recherche en Sciences de la Santé, Nanoro, Burkina Faso; Disease Control and Elimination Theme, Medical Research Council Unit, The Gambia at London School of Hygiene and Tropical Medicine (MRCG @ LSHTM), Banjul, The Gambia; Disease Control and Elimination Theme, Medical Research Council Unit, The Gambia at London School of Hygiene and Tropical Medicine (MRCG @ LSHTM), Banjul, The Gambia; Clinical Research Unit of Nanoro, Institut de Recherche en Sciences de la Santé, Nanoro, Burkina Faso

**Keywords:** intrapartum azithromycin, *Escherichia coli*, *Klebsiella pneumoniae*, bacterial carriage, antibiotic resistance

## Abstract

**Background:**

Limited data exist on the effects of intrapartum azithromycin on the prevalence of carriage and antibiotic resistance of Enterobacterales.

**Methods:**

We conducted a randomized trial in The Gambia and Burkina Faso where women received intrapartum azithromycin (2 g) or placebo. We determined the impact of treatment on the prevalence of carriage and antibiotic resistance of *Escherichia coli* and *Klebsiella pneumoniae* by analyzing rectal swabs (RS), nasopharyngeal swabs (NPS), breast milk, and rectovaginal swabs (RVS). Bacteria were isolated microbiologically; antibiotic susceptibility was confirmed with an E-test. Prevalence ratios (PRs) with 95% confidence intervals (CIs) were used for comparison between arms.

**Results:**

In infants, *E. coli* carriage in RS was lower in the intervention than in the placebo arm at day 6 (63.0% vs 75.2%; PR, 0.84; 95% CI, .75–.95) and day 28 (52.7% vs 70.4%; 0.75; 0.64–0.87) post-intervention. Prevalence of azithromycin-resistant *E. coli* was higher in the azithromycin arm at day 6 (13.4% vs 3.6%; 3.75; 1.83–7.69) and day 28 (16.4% vs 9.6%; 1.71; 1.05–2.79). For *K. pneumoniae*, carriage in RS was higher in the intervention than in the placebo arm at day 6 (49.6% vs 37.2%, 1.33; 1.08–1.64) and day 28 (53.6% vs 32.9%, 1.63; 1.31–2.03). Prevalence of azithromycin-resistant *K. pneumoniae* was higher in the azithromycin arm at day 28 (7.3% vs 2.1%; 3.49; 1.30–9.37). No differences were observed for other sample types.

**Conclusions:**

Intrapartum azithromycin decreased *E. coli* carriage but increased both *K. pneumoniae* carriage and azithromycin resistance in both bacteria. These data need to be considered together with efficacy results to balance the potential short- and long-term impact of the intervention.

***Clinical Trials Registration*.**  www.clinicaltrials.gov: NCT03199547.

## Teaser

We determined the effect of intrapartum azithromycin on carriage and antibiotic resistance of *Escherichia coli* and *Klebsiella pneumoniae*, major pathogens of neonatal sepsis. The intervention decreased *E. coli* and increased *K. pneumoniae* carriage. It increased azithromycin resistance in both bacteria.

## Graphical Abstract

**Graphical Abstract:**
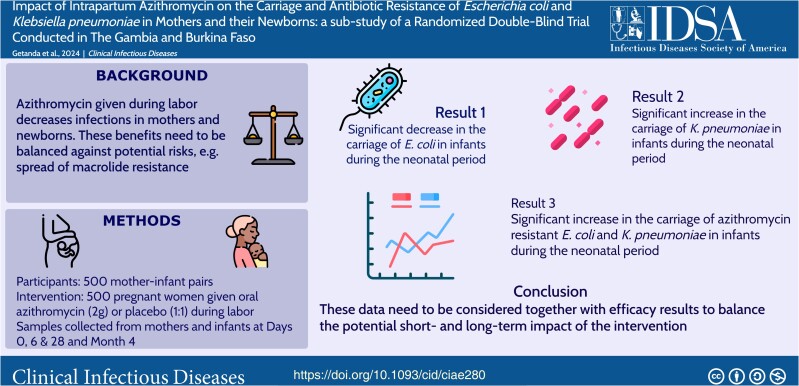
This graphical abstract is also available at Tidbit: https://tidbitapp.io/tidbits/impact-of-intrapartum-azithromycin-on-the-carriage-and-antibiotic-resistance-of-escherichia-coli-and-klebsiella-pneumoniae-in-mothers-and-their-newborns-a-sub-study-of-a-randomized-double-blind-trial-con-4ee9b8f1-72e6-47b1-8dcc-ba7b68c092ea?utm_campaign=tidbitlinkshare&utm_source=ITP

Efforts to reduce global neonatal mortality rates have led to a 50% decrease, from 36.6 to 17.5 per 1000 live births between 1990 and 2019 [1]. Nevertheless, progress varies across regions [2]. Over the same period, neonatal mortality rates in sub-Saharan Africa decreased by 26%, currently representing 43% of global neonatal deaths [1]. Neonatal sepsis, a major contributor to neonatal mortality [3], is often caused by *Staphylococcus aureus*, *Escherichia coli*, and *Klebsiella* spp., with varying prevalence in different African subregions [4, 5]. For example, *S. aureus* sepsis is more prevalent in West Africa, while *Klebsiella* spp. sepsis is more prevalent in Central and South Africa [4]. Maternal vaginal *S. aureus* colonization, which is correlated with neonatal colonization and subsequent disease, is estimated to be 16% in West Africa, 29% in Central Africa, and 2%–8% in East Africa [6–9].

Azithromycin, a second-generation macrolide antibiotic, exhibits broad-spectrum activity against gram-positive and some gram-negative bacteria [10]. Its oral administration results in rapid absorption reaching peak concentrations in blood or tissues within 2–3 hours [11], becoming a potential prophylactic antibiotic for preventing neonatal and maternal infections.

Two recent, double-blinded, randomized trials, PregnAnZI-2 and A-PLUS, explored the use of azithromycin to decrease neonatal sepsis and mortality across 9 African and Asian countries [12, 13]. Although no reduction in neonatal sepsis and mortality was observed, a significant impact in reducing maternal infections [12], including puerperal sepsis [13], was noted. The PregnAnZI-2 trial, conducted in West Africa, also reported a reduction in neonatal infections and a lower rate of prescribed antibiotics during the neonatal period [12].

Our earlier research showed that this intervention reduces gram-positive bacterial colonization in mothers and newborns throughout the neonatal period, including *S. aureus* [6]. Despite this, a temporary increase in azithromycin-resistant *S. aureus* that lasted between 1 and 12 months was observed [14]. Additionally, intrapartum azithromycin lowered carriage of *Streptococcus pneumoniae* and groups A and B *Streptococcus* without increasing antibiotic resistance [6, 15]. Data on the effect of intrapartum azithromycin on carriage and antibiotic resistance of 2 gram-negative bacteria that cause neonatal sepsis, *E. coli* and *K. pneumoniae*, are scarce [16]. It is important to evaluate the impact of the intervention on these 2 gram-negative bacteria due to their role in neonatal sepsis and their rising rates of multidrug resistance, which severely limits treatment options [17]. Our aim in this study was to determine the effect of intrapartum azithromycin on the prevalence of carriage and antibiotic resistance of *E. coli* and *K. pneumoniae* among mother–infant pairs from The Gambia and Burkina Faso.

## METHODS

### Overall Trial Design

PregnAnZI-2 was a phase 3, double-blind, placebo-controlled, randomized clinical trial that recruited 12 000 women in The Gambia and Burkina Faso to receive either oral azithromycin (2 g) or placebo during labor (ratio 1:1). Women aged 16+ years were consented during antenatal visits and enrolled in the trial after oral consent at study health facilities during labor [18].

### Study Sites

In The Gambia, women were recruited from 2 peri-urban government health facilities located close to the capital, Banjul. In Burkina Faso, women were recruited from 8 health facilities in rural central districts of Nanoro and Yako (Supplementary Figure 1).

### The Carriage Substudy

A subgroup of 250 mother–infant pairs per country participated in this substudy. They were enrolled in the trial between 23 January 2019 and 27 March 2020 in The Gambia and between 2 April 2019 and 8 April 2020 in Burkina Faso.

Biological samples were collected pre-intervention until 4 months post-intervention. A maternal nasopharyngeal swab (NPS) and rectovaginal swab (RVS) were collected during labor before the intervention. Within 4 hours after birth, an NPS and a rectal swab (RS) were collected from newborns. Additional samples were collected during household visits as follows: from mothers, NPS at day 6 and breast milk (BM) at day 6, day 28, and month 4, and from infants, NPS and RS at day 6, day 28, and month 4. For The Gambia, the last 2 sample collection time points were affected by the state of emergency declared in March 2020 due to the coronavirus disease 2019 pandemic [19].

### Sample Handling and Laboratory Methods

RVS were collected using a sterile cotton swab inserted 2–3 cm into the vagina and rotated in a circular motion for 5 seconds. The same swab was inserted 2–3 cm through the anal sphincter and rotated in a circular motion for 5 seconds. The latter procedure was done to collect RS from infants. Sample collection for NPS and BM samples was done as previously described [20]. Swabs were placed in a vial that contained skim milk-tryptone-glucose-glycerol transport medium in a cold box and transported to the laboratories within 8 hours. On arrival, samples were vortexed for 20 seconds and stored at −70°C for batch processing. Samples collected in Burkina Faso were shipped to The Gambia on dry ice and stored as described above.

### Identification of *E. coli* and *K. pneumoniae*

*Escherichia coli* and *K. pneumoniae* were isolated from mothers’ BM and RVS and newborns’ RS. In addition, *K. pneumoniae* was isolated from participants’ NPS (Supplementary Figure 2). Samples were thawed on ice and vortexed briefly. An aliquot of 50 μL dispensed onto MacConkey agar (Oxoid, UK) was streaked for selective isolation of *E. coli* and *K. pneumoniae* as previously described [16]. For *E. coli*, identification was done for each morphologically distinct suspected colony when more than 1 was available, and each was stored separately.

### Antibiotic Susceptibility

We performed disc diffusion on 3–5 well-isolated *E. coli* colonies or *K. pneumoniae* as previously described [16]. We tested for susceptibility to azithromycin and 9 other antibiotics (ampicillin, trimethoprim-sulfamethoxazole, gentamicin, ciprofloxacin, cefoxitin, ceftazidime, cefotaxime, amoxicillin-clavulanic acid, and meropenem). Production of extended-spectrum ß-lactamase (ESBL) was determined using the double-disc synergy diffusion test (Clinical and Laboratory Standards Institute) [21]. The minimum inhibitory concentration for all azithromycin-nonsusceptible isolates, 5% of azithromycin-susceptible isolates, all ESBL producers, and 2% of ESBL nonproducers was determined using E-test strips (Biomérieux, Marcy l’Etoile, France) per manufacturer's instructions. Antibiotic concentrations are included in prevalence tables. The CLSI lacks clinical break points for *E. coli* and *K. pneumoniae* azithromycin resistance; details of cutoffs are provided in Tables 2 and 3 [22, 23]. The strains *E. coli* American Type Culture Collection (ATCC) 25922 and *K. pneumoniae* ATCC 700603 were used as controls.

### Statistical Analyses

The prevalence of bacterial carriage and antibiotic resistance was compared between trial arms using prevalence ratios (PRs), with corresponding 95% confidence intervals (CIs). The Fisher exact test was used to obtain *P* values; *P* < .05 was considered significant. Stata version 18 was used for all analyses. In the main analysis, the total number of available samples was used as the denominator. When we identified more than 1 *E. coli* isolate per RVS and RS, to calculate prevalence of *E. coli* carriage at a particular site, we considered a participant a “carrier” if at least 1 *E. coli* was isolated from the sample. A sample was considered “resistant” for a specific antibiotic if at least 1 resistant *E. coli* isolate was present. For *K. pneumoniae*, only 1 isolate per sample was identified and tested for resistance. In addition, we determined the frequency of antibiotic resistance in infants’ RS for samples that were positive for *E. coli* and *K. pneumoniae*.

### Ethical Considerations

The joint Gambia Government/Medical Research Council Unit The Gambia Ethics Committee, the Comité d’Ethique pour la Recherche en Santé of Burkina Faso, and the LSHTM Ethics Committee approved the trial. Consent was sought concurrently for both the main trial and carriage substudy when pregnant women attended antenatal clinics.

## RESULTS

**Figure 1.**
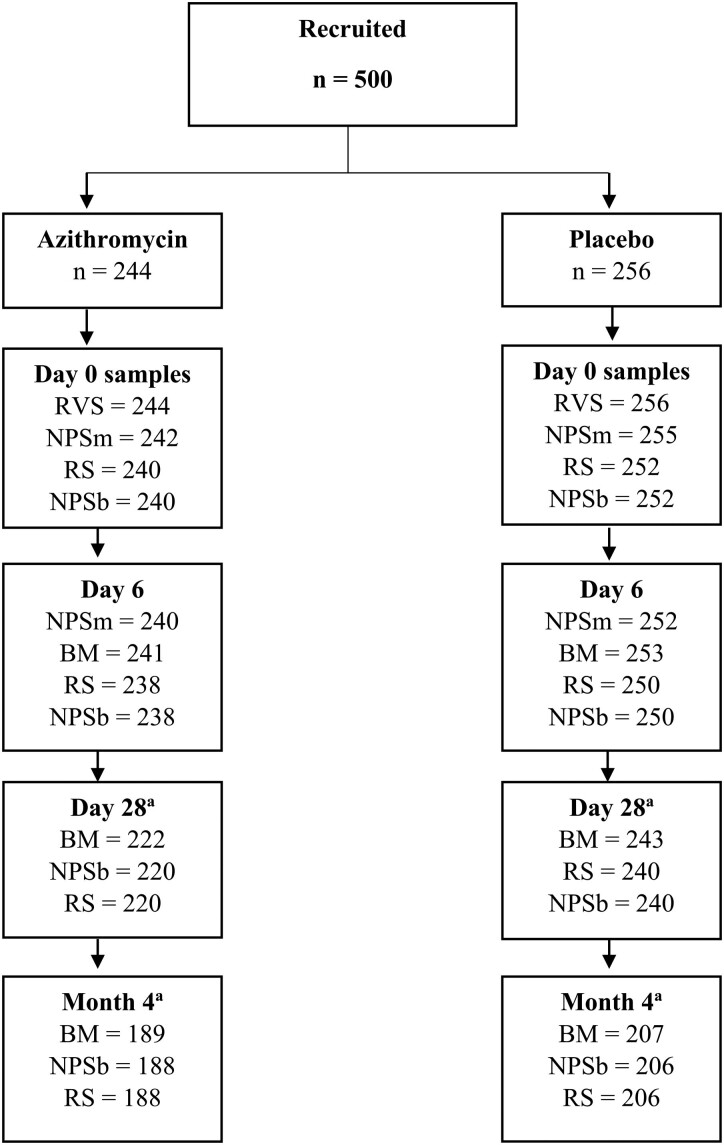
Study profile. Abbreviations: BM, breast milk; NPSb, nasopharyngeal swab (baby); NPSm, nasopharyngeal swab (mother); RS, rectal swab; RVS, rectovaginal swab. ^a^Sample collection affected by coronavirus disease 2019 disruptions.

Overall, 500 mother–infant pairs participated in this substudy, 250 from The Gambia and 250 from Burkina Faso (122 in azithromycin and 128 in placebo arm per country). The proportion of samples collected was >98% at day 0 and day 6, 92% at day 28, and 79% at month 4. Details of samples available at each time point are provided in Figure 1 (trial profile). Baseline characteristics of study arms are shown in Table 1.

**Table 1.** Baseline Characteristics

Mothers	Azithromycin	Placebo
	n = 244	n = 256
Country, n (%)		
The Gambia	122 (50.0)	128 (50.0)
Burkina Faso	122 (50.0)	128 (50.0)
Age,^a^ median (IQR), y	26.0 (21.0–31.0)	26.0 (21.0–30.0)
Ethnicity, n (%)		
Mandinka	47 (19.3)	50 (19.5)
Wollof	20 (8.2)	20 (7.8)
Jola	14 (5.7)	16 (6.3)
Fula	21 (8.6)	19 (7.4)
Mossi	115 (47.1)	120 (46.9)
Gourounsi	5 (2.0)	7 (2.7)
Peulh	2 (0.8)	1 (0.4)
Other	20 (8.2)	23 (9.0)
Season of delivery,^b^ n (%)		
Dry (Nov–May)	137 (56.1)	134 (52.3)
Wet (June–Oct)	106 (43.4)	120 (46.9)
Mode of delivery, n (%)		
Vaginal	238 (97.5)	251 (98.0)
Cesarean	6 (2.5)	5 (2.0)
Multiple pregnancy, n (%)	3 (1.2)	2 (0.8)
Newborns	n = 244	n = 256
Sex, n (%)		
Female	120 (49.2)	121 (47.3)
Male	124 (50.8)	135 (52.7)
Birth weight,^c^ median (IQR), kg	3.0 (2.8–3.3)	3 (2.8–3.35)

Abbreviation: IQR, interquartile range.

^a^Age missing for n = 52.

^b^Season of delivery missing for n = 3.

^c^Birth weight missing for n = 1.

### Prevalence of Carriage and Azithromycin Resistance of *E. coli*

#### Study Women

For pre-intervention RVS, prevalence of *E. coli* carriage was similar in azithromycin and placebo arms (68.9% and 67.6%, respectively; Table 2). Prevalence of carriage of azithromycin-resistant isolates was low and ranged from 2.7% to 4.5% (Table 2). For post-intervention samples, there were no differences between arms in the prevalence of carriage of *E. coli* or azithromycin-resistant *E. coli* in BM at any time point (Table 2). Analyses stratified by country are provided in Supplementary Tables 1*A* and 1*B*.

**Table 2.** Prevalence of *Escherichia coli* Carriage and Azithromycin Resistance in Different Biological Samples From Women and Their Infants

	Prevalence of Carriage				Prevalence of Resistance			
	AZI^a^ n/N (%)	Placebo n/N (%)	PR (95% CI)	*P* Value	AZI^a^ n/N (%)	Placebo n/N (%)	PR (95% CI)	*P* Value
Women: rectovaginal swab samples								
Day 0^b^	168/244 (68.9)	173/256 (67.6)	1.02 (.91–1.15)	.773	11/244 (4.5)	7/256 (2.7)	1.66 (.65–4.20)	.341
Women: breast milk samples								
Day 6	9/241 (3.7)	8/253 (3.2)	1.18 (.46–3.01)	.808	4/241 (1.7)	0/253	…	.056
Day 28	1/222 (0.5)	2/243 (0.8)	0.55 (.05–5.99)	1.000	0/222	1/243 (0.4)	…	1.000
Month 4	0/189	0/207	…	…	0/189	0/207	…	…
Children: rectal swab samples								
Day 0^c^	8/240 (3.3)	14/252 (5.56)	0.60 (.26–1.41)	.279	1/240 (0.42)	0/252	…	.487
Day 6	**150/238 (63.0)**	**188/250 (75.2)**	**0.84 (.75–.95)**	**.006**	**32/238 (13.4)**	**9/250 (3.6)**	**3.75 (1.83–7.69)**	**<.001**
Day 28	**116/220 (52.7)**	**169/240 (70.4)**	**0.75 (.64–.87)**	**<.001**	**36/220 (16.4)**	**23/240 (9.6)**	**1.71 (1.05–2.79)**	**.036**
Month 4	140/188 (74.5)	160/206 (77.7)	0.96 (.86–1.07)	.479	31/188 (16.5)	23/206 (11.2)	1.48 (.89–2.44)	.143

Isolates with minimum inhibitory concentrations ≥32 μg/mL considered resistant based on AZI epidemiological cutoff values and limited clinical data for other Enterobacterales *P* values from the Fisher exact test. Values in bold indicate statistically significant values *p* < .05.

Abbreviations: AZI, azithromycin; CI, confidence interval; PR, prevalence ratio.

^a^Antibiotic concentration: ^a^azithromycin (0.016–256 μg/mL).

^b^Samples collected at day 0, pre-intervention.

^c^Samples collected at day 0, post-intervention.

#### Study Infants

**Figure 2.**
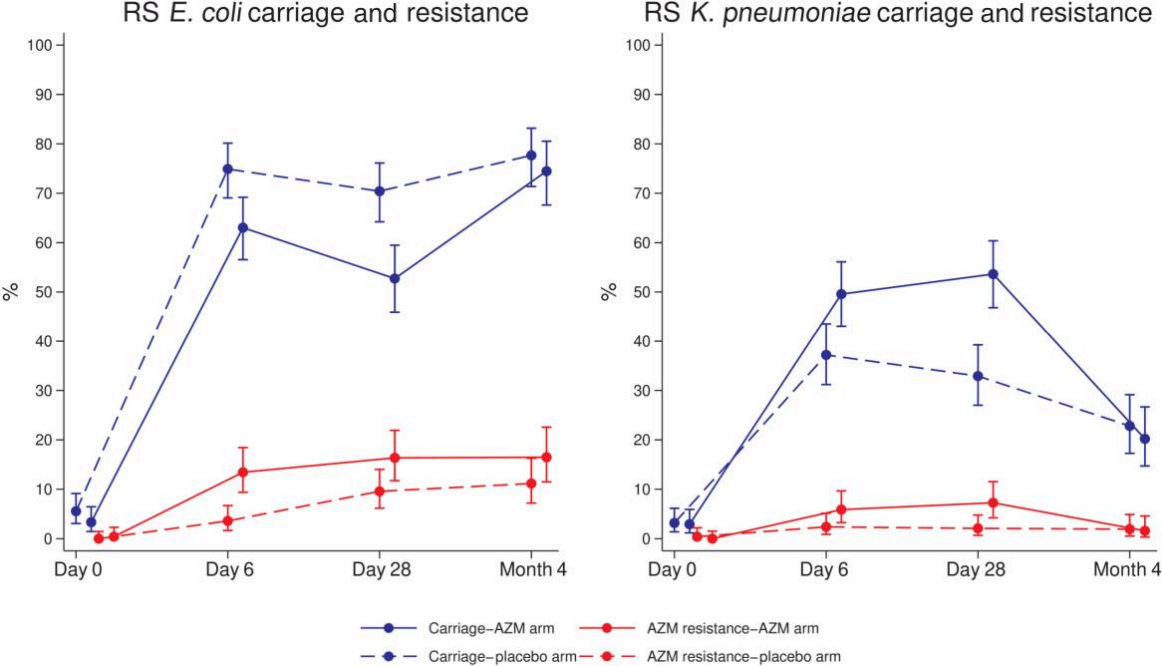
RS *Escherichia coli* and *Klebsiella pneumoniae* carriage and azithromycin resistance. Abbreviations: AZM, azithromycin; RS, rectal swab.

Prevalence of *E. coli* carriage in infants’ RS samples was lower in the azithromycin arm compared with placebo at day 6 (63.0% vs 75.2%; PR, 0.84; CI, .75–.95; *P* = .006) and day 28 (52.7% vs 70.4%; 0.75; 0.64–0.87; *P* < .001; Table 2, Figure 2). Prevalence of azithromycin-resistant *E. coli* in the azithromycin arm was significantly higher at day 6 (13.4% vs 3.6%; 3.75; 1.83–7.69; *P* < .001) and day 28 (16.4% vs 9.6%; 1.71; 1.05–2.79; *P* = .036; Table 2, Figure 2). The frequency of azithromycin resistance among *E. coli* isolated from RS was higher in the azithromycin arm at day 6 and day 28 (Supplementary Table 2). Analyses stratified by country are provided in Supplementary Tables 1*A* and 1*B*.

### Prevalence of *E. coli* Resistance to Other Antibiotics

#### Study Women

For pre-intervention RVS and post-intervention BM, there were no differences between arms in the prevalence of *E. coli* resistant to ampicillin, trimethoprim-sulfamethoxazole, gentamicin, and ciprofloxacin. For pre-intervention RVS, there was higher prevalence of ESBL carriage (2.0% vs 0%, *P* = .027) in the azithromycin arm compared with placebo (Supplementary Tables 3*A*, 3*B*, and 3*C*). There was no resistance to meropenem, and cefoxitin resistance was low.

#### Study Infants

For infants’ RS, prevalence of carriage of *E. coli* resistant to ampicillin at day 6 (46.2% vs 58.4%; 0.80; 0.67–0.94; *P* = .009) and day 28 (44.1% vs 59.6%; 0.74; 0.62–0.89; *P* = .001) was lower in the azithromycin arm (Supplementary Table 3*A*, Supplementary Figure 3). Prevalence of carriage of *E. coli* resistant to trimethoprim-sulfamethoxazole was lower in the azithromycin arm at day 6 (45.4% vs 57.6%; 0.79; 0.66–0.94; *P* = .009) and day 28 (42.3% vs 57.1%; 0.74; 0.61–0.89; *P* = .002; Supplementary Table 3*A*, Supplementary Figure 3). Prevalence of carriage of *E. coli* resistant to cefoxitin was lower at day 28 (0% vs 2.9%, *P* = .016) in the azithromycin arm (Supplementary Table 3*C*, Supplementary Figure 3). Prevalence of *E. coli* resistant to gentamicin, ciprofloxacin, and ESBL carriage was similar between arms (Supplementary Tables 3*B* and 3*C*, Supplementary Figure 3). No meropenem-resistant *E. coli* was detected. Details of frequency of antibiotic resistance among *E. coli* isolated from RS are provided in Supplementary Table 2.

### Prevalence of Carriage and Azithromycin Resistance of *K. pneumoniae*

#### Study Women

Prevalence of *K. pneumoniae* carriage was similar between arms for all samples and time points (Table 3). Prevalence of azithromycin-resistant isolates before and after the intervention was low and similar between arms (0.4% vs 1.6%; Table 3). Analyses stratified by country are provided in Supplementary Tables 4*A* and 4*B*.

**Table 3.** Prevalence of *Klebsiella pneumoniae* Carriage and Azithromycin Resistance in Different Biological Samples From Women and Their Infants

	Prevalence of Carriage				Prevalence of Resistance			
	AZI^a^ n/N (%)	Placebo n/N (%)	PR (95% CI)	*P* Value	AZI^a^ n/N (%)	Placebo n/N (%)	PR (95% CI)	*P* Value
Women: rectovaginal swab samples								
Day 0^b^	67/244 (27.5)	68/256 (26.6)	1.03 (.77–1.38)	.841	1/244 (0.4)	4/256 (1.6)	0.26 (.03–2.33)	.373
Women: nasopharyngeal swab samples								
Day 0^b^	4/243 (1.6)	10/254 (3.9)	0.42 (.13–1.32)	.175	0/243	1/254 (0.4)	…	1.000
Day 6	8/240 (3.3)	2/252 (0.8)	4.2 (.90–19.58)	.057	0/240	0/252	…	…
Women: breast milk samples								
Day 6	12/241 (5.0)	12/253 (4.7)	1.05 (.48–2.29)	1.000	3/241 (1.2)	1/253 (0.4)	3.15 (.33–30.07)	.362
Day 28	12/222 (5.4)	7/243 (2.9)	1.88 (.76–4.70)	.241	1/222 (0.5)	0/243	…	.476
Month 4	5/189 (2.6)	3/207 (1.4)	1.83 (.44–7.53)	.487	0/189	0/207	…	…
Children: rectal swab samples								
Day 0^c^	7/240 (2.9)	8/252 (3.2)	0.92 (.34–2.49)	1.000	0/240	1/252 (0.4)	…	1.000
Day 6	**118/238 (49.6)**	**93/250 (37.2)**	**1.33 (1.08–1.64)**	**.006**	14/238 (5.9)	6/250 (2.4)	2.45 (.96–6.27)	.067
Day 28	**118/220 (53.6)**	**79/240 (32.9)**	**1.63 (1.31–2.03)**	**<.001**	**16/220 (7.3)**	**5/240 (2.1)**	**3.49 (1.30–9.37)**	**.012**
Month 4	38/188 (20.2)	47/206 (22.8)	0.89 (.61–1.29)	.543	3/188 (1.6)	4/206 (1.9)	0.82 (.19–3.62)	1.000
Children: nasopharyngeal swab samples								
Day 0^c^	1/240 (0.4)	0/252	…	.487	0/240	0/252	…	…
Day 6	20/238 (8.4)	16/250 (6.4)	1.32 (.70–2.49)	.393	0/238	0/250	…	…
Day 28	4/220 (1.8)	5/240 (2.1)	0.87 (.24–3.20)	1.000	0/220	0/240	…	…
Month 4	1/188 (0.5)	0/206	…	.477	0/188	0/206	…	…

Isolates with minimum inhibitory concentrations ≥32 μg/mL considered resistant based on AZI epidemiological cutoff values and limited clinical data for other Enterobacterales *P* values from the Fisher exact test. Values in bold indicate statistically significant values *p* <.05.

Abbreviations: AZI, azithromycin; CI, confidence interval; PR, prevalence ratio.

^a^Antibiotic concentration: ^a^azithromycin (0.016–256 μg/mL).

^b^Samples collected at day 0, pre-intervention.

^c^Samples collected at day 0, post-intervention.

#### Study Infants

Prevalence of *K. pneumoniae* carriage in RS was higher in the azithromycin arm at day 6 (49.6% vs 37.2%; 1.33; 1.08–1.64; *P* = .006) and day 28 (53.6% vs 32.9%; 1.63; 1.31–2.03; *P* < .001; Table 3, Figure 2). For azithromycin-resistant *K. pneumoniae* in RS, study arms were different at day 28 (7.3% vs 2.1%; 3.49; 1.30–9.37; *P* = .012) in the azithromycin arm vs placebo (Table 3, Figure 2). Details of frequency of azithromycin resistance among *K. pneumoniae* isolated from RS are provided in Supplementary Table 5. For NPS, no differences between arms were found for prevalence of *K. pneumoniae* carriage nor azithromycin resistance (Table 3). Analyses stratified by country are provided in Supplementary Tables 4*A* and 4*B*.

### Prevalence of *K. pneumoniae* Resistance to Other Antibiotics

#### Study Women

For pre-intervention maternal RVS and post-intervention BM, there were no differences between arms in the prevalence of *K. pneumoniae* resistant to trimethoprim-sulfamethoxazole, gentamicin, ciprofloxacin, and ESBL carriage. For pre-intervention maternal RVS, prevalence of *K. pneumoniae* resistant to cefoxitin was higher in the azithromycin arm (2.0% vs 0%; *P* = .027; Supplementary Table 6*C*). No resistance to meropenem was detected. For maternal NPS, resistance to all antibiotics was either absent or low.

#### Study Infants

In RS, resistance to trimethoprim-sulfamethoxazole (23.2% vs 8.8%; 2.65; 1.65–4.26; *P* < .001), gentamicin (10.5% vs 5.0%; 2.09; 1.07–4.10; *P* = .034), ciprofloxacin (15.5% vs 5.8%; 2.65; 1.46–4.80; *P* = .001), and ESBL carriage (9.5% vs 3.3%; 2.86; 1.30–6.33; *P* = .007) at day 28 was higher in the azithromycin arm (Supplementary Tables 6*A*, 6*B*, and 6*C*; Supplementary Figure 3); resistance to cefoxitin was low (Supplementary Table 6*C*) with no resistance to meropenem. The frequency of antibiotic resistance among *K. pneumoniae* isolated from RS samples is listed in Supplementary Table 5. In NPS, resistance to all tested antibiotics was either low or absent.

## DISCUSSION

Clinical trials have shown that prophylactic intrapartum azithromycin decreases maternal and neonatal infections [12, 13]. It is important, therefore, to evaluate the effect of this intervention on bacterial colonization and antimicrobial resistance. Previous studies have shown that the intervention decreases carriage of the main gram-positive bacteria that cause sepsis in mothers and newborns, with very little effect on azithromycin resistance [6, 15]. In this study, azithromycin reduced *E. coli* carriage and increased *K. pneumoniae* carriage, predominantly in infants’ RS. The intervention increased the carriage of azithromycin-resistant isolates for both bacteria. It simultaneously decreased the carriage of *E. coli* resistant to other antibiotics and increased the carriage of *K. pneumoniae* resistant to other antibiotics.

In a previous trial conducted in The Gambia following the same design, azithromycin (2 g) remained in the maternal BM for at least 4 weeks post-intervention, reaching peak levels on day 6 [24]. The substantial concentration of azithromycin transferred to infants, coupled with the impact on maternal carriage, likely explains the effects observed in infants in this study. However, such an effect on RS carriage of *E. coli* only lasted the neonatal period. These findings are consistent with the effect of azithromycin mass drug administration that reduced the short-term risk of diarrhea in infants aged 2–59 months, diarrheagenic *E. coli* being a major cause of diarrhea at this age [25–27]. In vitro experiments have also shown that azithromycin is efficacious against certain strains of pathogenic *E. coli* [28]. Moreover, azithromycin can effectively reduce bacterial shedding in patients with Shiga toxin–producing enteroaggregative *E. coli* and travelers’ diarrhea caused by enterotoxigenic *E. coli* [29, 30]. Azithromycin use, particularly in mass drug administration campaigns, increased carriage of azithromycin-resistant *E. coli* [31–33], as observed for RS in our study. We observed similar results in a previous study that used vaginal samples collected 8–10 days after the intervention [16]. The observation that at 4 months post-intervention the carriage of azithromycin-resistant *E. coli* is similar between study arms suggests that the effect on azithromycin resistance is probably waning with decreased drug pressure.

The increased azithromycin resistance was not matched by an increased *E. coli* resistance to other antibiotics. On the contrary, infants whose mothers had taken intrapartum azithromycin had a lower prevalence of ampicillin, trimethoprim-sulfamethoxazole, and cefoxitin-resistant *E. coli* isolates. There are 2 plausible explanations for this. First, the lower use of prescribed antibiotics in infants from the azithromycin arm due to lower rates of infections observed during the trial [12] may have resulted in a lower selective pressure and thus lower resistance to common antibiotics. Ampicillin and trimethoprim-sulfamethoxazole are broad-spectrum antibiotics often used for the treatment of respiratory, gastrointestinal, and urinary tract infections in West Africa [34, 35]. Second, lower overall prevalence of *E. coli* carriage in RS would translate to a lower prevalence of carriage of isolates resistant to other antibiotics. The similar frequency of *E. coli* isolates resistant to the different antibiotics in both arms would support this last hypothesis. This decreased prevalence of carriage of *E. coli*–resistant isolates is an encouraging result that needs to be interpreted considering further evaluation of the overall effects on the microbiome and resistome by the intervention.

We previously showed a higher carriage of *K. pneumoniae* isolated in BM samples collected after azithromycin treatment [16]. In this study, intrapartum azithromycin increased the risk of *K. pneumoniae* carriage in infants’ RS. The strong effect of azithromycin on gram-positive bacteria [6, 15] and certain gram-negative bacteria, as observed with *E. coli* here, may have advantaged *K. pneumoniae* at these body sites. Overgrowth of certain bacterial species after using broad-spectrum antibiotics has been reported. A study that investigated the effect of early-life antibiotics on the developing infant gut showed that antibiotic-treated infants had a higher abundance of *Klebsiella* spp. [36]. Nevertheless, this higher *K. pneumoniae* carriage did not increase the incidence of *K. pneumoniae* sepsis in our PregnAnZI-2 trial [12] or the A-PLUS trial (conducted in 7 low- and middle-income countries) [13].

In our study, it is possible that the higher prevalence of *K. pneumoniae* carriage resulted in a high carriage of azithromycin-resistant strains in RS, as we previously showed for BM [16]. Indeed, the time of the highest carriage of *K. pneumoniae* (day 28) coincides with that of the highest prevalence of azithromycin-resistant *K. pneumoniae* isolates in infants’ RS. In addition, day 28 was also the time point with higher resistance to other tested antibiotics, possibly caused by the same phenomenon. In support of this, we observed a similar trend in the frequency of resistant isolates for all antibiotics, including azithromycin. The production of ESBL in Enterobacterales mediates simultaneous acquisition of resistance to other classes of antibiotics because resistance genes may be located on the same mobile genetic elements [37] and could have contributed to the increased resistance to other antibiotics in *K. pneumoniae* at day 28.

This study had some limitations. Although we have shown the effect of the intervention on resistance to azithromycin and other antibiotics in *E. coli* and *K. pneumoniae*, we could not ascertain the mechanisms of resistance involved. This requires genomic evaluation to complement phenotypic observations. Also, despite the reduction of *E. coli* carriage following intrapartum azithromycin, it was not possible to determine whether such reduction is beneficial as we have not distinguished between pathogenic and nonpathogenic *E. coli*.

## CONCLUSIONS

Intrapartum azithromycin decreases carriage of *E. coli* and increases carriage of *K. pneumoniae* in the gut of neonates. The intervention also increases carriage of azithromycin-resistant *E. coli* and *K. pneumoniae* isolates, a potential threat to the spread of such resistance to the community. Conversely, this intervention may decrease resistance to other commonly used antibiotics such as ampicillin or trimethoprim-sulfamethoxazole in *E. coli* either because it decreases carriage or antibiotic prescription. These results need to be considered when evaluating the overall impact of the use of azithromycin to prevent maternal, neonatal, and infant infections.

## Supplementary Data

Supplementary materials are available at *Clinical Infectious Diseases* online. Consisting of data provided by the authors to benefit the reader, the posted materials are not copyedited and are the sole responsibility of the authors, so questions or comments should be addressed to the corresponding author.

## Supplementary Material

ciae280_Supplementary_Data

## Notes

***Author Contributions.*** A. R., H. T., and U. D. A. conceived the trial. A. R. and P. G. designed this substudy. P. G. wrote the manuscript, and A. R. contributed significantly to its revision. B. C., J. D. B., I. J., S. D., E. S., M. B., K. K., A .C., H. M., and B. K. J. S. were involved in the adaptation of the field and laboratory work. A. B., B. H., and B. S. critically reviewed the manuscript. E. N. was involved in data management. C. B. and T. R. assisted with the statistical analyses and critically reviewed the manuscript.

***Acknowledgments.*** The authors thank the members of the PregnAnZI-2 trial, field, laboratory, and data teams and all staff of the clinical microbiology laboratory of The Gambia at London School of Hygiene and Tropical Medicine (MRCG @ LSHTM). We also sincerely thank all participants for agreeing to take part in the study. The authors acknowledge the following PregnAnZI-2 carriage study team members: Fatoumata Sillah, Nathalie Beloum, Usman N. Nakakana, Madikoi Danso, Joquina C. Jones, Shashu Graves, Edrissa Sabally, Siaka Badjie, Sulayman Bah, Omar B. Jarra, and Abdoulie Suso.

***Financial support***. This work was supported by the Medical Research Council Unit The Gambia at the London School of Hygiene and Tropical medicine MRCG@LSHTM PhD fellowship. The main trial was funded by UK Research and Innovation under the Joint Global Health Trials Scheme, reference MC_EX_MR/P006949/1, and the Bill & Melinda Gates Foundation, reference INV-000253.
