# Supplementary material for: Impact of Intrapartum Azithromycin on the Carriage and Antibiotic Resistance of Escherichia coli and Klebsiella pneumoniae in Mothers and Their Newborns: A Substudy of a Randomized, Double-Blind Trial Conducted in The Gambia and Burkina Faso
Source: Clin Infect Dis. 2024 May 16;79(6):1338–45. doi: 10.1093/cid/ciae280 (PMC11650870; doi:10.1093/cid/ciae280)

**Supplementary Table 1a: Prevalence of *E. coli* carriage** **in different biological samples from women and their infants (stratified by country)**

|  | **The Gambia** | | | | **Burkina Faso** | | | |
| --- | --- | --- | --- | --- | --- | --- | --- | --- |
|  | **Prevalence of carriage** | | | | **Prevalence of carriage** | | | |
|  | **AZM**  **n/N (%)** | **Placebo n/N (%)** | **PR (95% CI)** | **p-value** | **AZM**  **n/N (%)** | **Placebo**  **n/N (%)** | **PR (95% CI)** | **p-value** |
| **Women - rectovaginal swab samples** | | | | | | | | |
| **Day 0^1^** | 80/122 (65.6) | 90/128 (70.3) | 0.94 (0.79-1.11) | 0.498 | 88/122 (72.1) | 83/128  (64.8) | 1.11 (0.94-1.32) | 0.224 |
| **Women - breast milk samples** | | | | | | | | |
| **Day 6** | 8/120  (6.7) | 5/125  (4) | 1.67 (0.56-4.95) | 0.403 | 1/121  (0.8) | 3/128  (2.3) | 0.35 (0.04-3.34) | 0.623 |
| **Day 28** | 1/101 (1.0) | 1/116 (0.8) | 1.15 (0.07-18.12) | 1.000 | 0/121 | 1/127  (0.8) | - | 1.000 |
| **Month 4** | 0/69 | 0/83 | - | - | 0/120 | 0/124 | - | - |
| **Children - rectal swab samples** | | | | | | | | |
| **Day 0^2^** | 5/120  (4.2) | 12/127 (9.4) | 0.44 (0.16-1.22) | 0.133 | 3/120  (2.5) | 2/125 (1.6) | 1.56 (0.27-9.19) | 0.679 |
| **Day 6** | 83/119 (69.7) | 95/122 (77.9) | 0.90 (0.78-1.05) | 0.194 | **67/119 (56.3)** | **93/128**  **(72.7)** | **0.77 (0.64-0.94)** | **0.008** |
| **Day 28** | **57/101 (56.4)** | **87/114 (76.3)** | **0.74 (0.61-0.90)** | **0.002** | **59/119 (49.6)** | **82/126**  **(65.1)** | **0.76 (0.61-0.95)** | **0.020** |
| **Month 4** | 54/69 (82.6) | 64/82 (85.4) | 1.00 (0.85-1.19) | 1.000 | 86/119 (72.3) | 96/124  (77.4) | 0.93 (0.81-1.08) | 0.378 |

^1^Samples collected at Day 0, pre-intervention ^2^Samples collected at Day 0, post-intervention p-values from Fisher’s exact test

**Supplementary Table 1b: Prevalence of *E. coli* azithromycin resistance in different biological samples from women and their infants (stratified by country)**

|  | **The Gambia** | | | | **Burkina Faso** | | | |
| --- | --- | --- | --- | --- | --- | --- | --- | --- |
|  | **Prevalence of azithromycin^a^ resistance** | | | | **Prevalence azithromycin^a^ resistance** | | | |
|  | **AZM**  **n/N (%)** | **Placebo n/N (%)** | **PR (95% CI)** | **p-value** | **AZM**  **n/N (%)** | **Placebo n/N (%)** | **PR (95% CI)** | **p-value** |
| **Women - rectovaginal swab samples** | | | | | | | | |
| **Day 0^1^** | n=122 | n=128 |  |  | n=122 | n=128 |  |  |
|  | 6/122  (4.9) | 3/128  (2.3) | 2.12 (0.54-8.27) | 0.323 | 5/122  (4.1) | 4/128 (3.1) | 1.31 (0.36-4.77) | 0.744 |
| **Women - breast milk samples** | | | | | | | | |
| **Day 6** | n=120 | n=125 |  |  | n= 121 | n= 128 |  |  |
|  | 4/120  (3.3) | 0/125 | - | 0.056 | 0/121 | 0/128 | - | - |
| **Day 28** | n=101 | n=116 |  |  | n= 121 | n= 127 |  |  |
|  | 0/101 | 1/116  (0.8) | - | 1.000 | 0/121 | 0/127 | - | - |
| **Month 4** | n=69 | n=83 |  |  | n= 120 | n= 124 |  |  |
|  | 0/69 | 0/83 | - | - | 0/120 | 0/124 | - | - |
| **Children - rectal swab samples** | | | | | | | | |
| **Day 0^2^** | 0/120 | 0/127 | - |  | 1/120  (0.8) | 0/125 | - | 0.490 |
| **Day 6** | **16/119 (13.4)** | **2 /122**  **(1.6)** | **8.27 (1.94-35.19)** | **<0.001** | **16/119**  **(13.4)** | **7/128 (5.5)** | **2.46 (1.05-5.77)** | **0.047** |
| **Day 28** | 17/101 (16.8) | 11/114 (9.6) | 1.74(0.86-3.55) | 0.155 | 19/119  (16.0) | 12/126 (9.5) | 1.68 (0.85-3.30) | 0.178 |
| **Month 4** | **11/69 (15.9)** | **3/82**  **(3.7)** | **4.36 (1.27-14.99)** | **0.012** | 20/119  (16.8) | 20/124  (16.1) | 1.04 (0.59-1.84) | 1.000 |

^a^Antibiotic concentration: ^a^azithromycin (0.016-256µg/mL), ^1^Samples collected at Day 0, pre-intervention ^2^Samples collected at Day 0, post-intervention p-values from Fisher’s exact test

**Supplementary Table 2: Frequency of antibiotic resistance in *E. coli* from rectal swab (RS) samples from infants (*E. coli* positive samples as denominator)**

| **Prevalence of *E. coli* azithromycin resistance** | | | | | **Prevalence of *E. coli* ampicillin resistance** | | | |
| --- | --- | --- | --- | --- | --- | --- | --- | --- |
|  | **AZM**  **n/N (%)** | **Placebo n/N (%)** | **PR (95% CI)** | **p-value** | **AZM**  **n/N (%)** | **Placebo**  **n/N (%)** | **PR (95% CI)** | **p-value** |
| **Day 0^2^** | 1/8  (12.5) | 0/14 | - | 0.364 | 5/8  (62.5) | 9/14  (64.3) | 0.97 (0.50-1.89) | 1.000 |
| **Day 6** | **32/150 (21.3)** | **9/188**  **(4.8)** | **4.46 (2.20 - 9.04)** | **< 0.001** | 110/150  (73.3) | 146/188  (77.7) | 0.94 (0.83-1.07) | 0.374 |
| **Day 28** | **36/116 (31.0)** | **23/169 (13.6)** | **2.28 (1.43 - 3.64)** | **< 0.001** | 97/116  (83.6) | 143/169  (84.6) | 0.99 (0.89-1.10) | 0.869 |
| **Month 4** | 31/140 (22.1) | 23/160 (14.4) | 1.54 (0.94 - 2.51) | 0.098 | 124/140  (88.6) | 138/160  (86.3) | 1.03 (0.94-1.12) | 0.604 |
| **Prevalence of *E. coli* trimethoprim-sulfamethoxazole resistance** | | | | | **Prevalence of *E. coli* gentamicin resistance** | | | |
| **Day 0^2^** | 2/8  (25.0) | 8/14  (57.1) | 0.44 (0.12-1.58) | 0.204 | 2/8  (25.0) | 0/14 | - | 0.121 |
| **Day 6** | 108/150  (72.0) | 144/188  (76.6) | 0.94 (0.83-1.07) | 0.379 | 12/150  (8.0) | 11/188  (5.9) | 1.37 (0.62-3.01) | 0.516 |
| **Day 28** | 93/116  (80.2) | 137/169  (81.1) | 0.99 (0.88-1.11) | 0.879 | 12/116  (10.3) | 19/169  (11.2) | 0.92 (0.46-1.82) | 0.849 |
| **Month 4** | 115/140  (82.1) | 133/160  (83.1) | 0.99 (0.89-1.10) | 0.879 | 17/140  (12.1) | 12/160  (7.5) | 1.62 (0.80-3.27) | 0.240 |
| **Prevalence of *E. coli* ciprofloxacin resistance** | | | | | **Prevalence of *E. coli* cefoxitin resistance** | | | |
| **Day 0^2^** | 1/8  (12.5) | 1/14  (7.1) | 1.75 (0.13-24.33) | 1.000 | 2/8  (25.0) | 0/14 | - | 0.121 |
| **Day 6** | 23/150  (15.3) | 27/188  (14.4) | 1.07 (0.64-1.78) | 0.878 | 1/150  (0.7) | 0/188 | - | 0.444 |
| **Day 28** | 33/116  (28.4) | 35/169  (20.7) | 1.37 (0.91-2.08) | 0.157 | **0/116** | **7/162**  **(4.3)** | **-** | **0.044** |
| **Month 4** | 45/140  (32.1) | 53/160  (33.1) | 0.97 (0.70-1.34) | 0.902 | 3/140  (2.1) | 2/160  (1.3) | 1.71 (0.29-10.11) | 0.667 |
| **Prevalence of *E. coli* ESBL carriage** | | | | |  | | | |
| **Day 0^2^** | 0/8 | 0/14 | - | - |  |  |  |  |
| **Day 6** | 7/150  (4.7) | 11/188  (5.9) | 0.80 (0.32-2.01) | 0.808 |  |  |  |  |
| **Day 28** | 12/116  (10.3) | 16/169  (9.5) | 1.09 (0.54-2.22) | 0.841 |  |  |  |  |
| **Month 4** | 17/140  (12.1) | 22/160  (13.8) | 0.88 (0.49-1.59) | 0.733 |  |  |  |  |

Antibiotic concentrations as shown in Supplementary Tables 1b, 3a, 3b and 3c

^2^Samples collected at Day 0, post-intervention

p-values from Fisher’s exact test

**Supplementary Table 3a:** **Prevalence of *E. coli* ampicillin and trimethoprim-sulfamethoxazole resistance in different biological samples from women and their infants**

|  | **Prevalence of *E. coli* ampicillin^a^ resistance** | | | | **Prevalence of *E. coli* trimethoprim- sulfamethoxazole^b^ resistance** | | | |
| --- | --- | --- | --- | --- | --- | --- | --- | --- |
|  | **AZM**  **n/N (%)** | **Placebo n/N (%)** | **PR (95% CI)** | **p-value** | **AZM**  **n/N (%)** | **Placebo n/N (%)** | **PR (95% CI)** | **p-value** |
| **Women - rectovaginal swab samples** | | | | | | | | |
| **Day 0^1^** | 91/244  (37.3) | 87/256  (34.0) | 1.10 (0.87-1.39) | 0.455 | 112/244  (45.9) | 119/256 (46.5) | 0.99 (0.82-1.20) | 1.000 |
|  |  |  |  |  |  |  |  |  |
| **Women - breast milk samples** | | | | | | | | |
| **Day 6** | 8/241  (3.3) | 5/253 (2.0) | 1.68 (0.56-5.06) | 0.408 | 8/241  (3.3) | 4/253 (1.6) | 2.10 (0.64-6.88) | 0.251 |
| **Day 28** | 0/122 | 2/243 (0.8) | - | 0.500 | 0/222 | 2/243 (0.8) | - | 0.500 |
| **Month 4** | 0/189 | 0/207 | - | - | 0/189 | 0/207 | - | - |
| **Children - rectal swab samples** | | | | | | | | |
| **Day 0^2^** | 5/240  (2.1) | 9/252 (3.6) | 0.59 (0.20-1.72) | 0.420 | 2/240  (0.8) | 8/252 (3.2) | 0.26 (0.06-1.23) | 0.107 |
| **Day 6** | **110/238**  **(46.2)** | **146/250 (58.4)** | **0.80 (0.67-0.94)** | **0.009** | **108/238**  **(45.4)** | **144/250**  **(57.6)** | **0.79 (0.66-0.94)** | **0.009** |
| **Day 28** | **97/220**  **(44.1)** | **143/240**  **(59.6)** | **0.74 (0.62-0.89)** | **0.001** | **93/220**  **(42.3)** | **137/240 (57.1)** | **0.74 (0.61-0.89)** | **0.002** |
| **Month 4** | 124/188  (66.0) | 138/206 (67.0) | 0.99 (0.86-1.13) | 0.832 | 115/188  (61.2) | 133/206 (64.6) | 0.95 (0.81-1.10) | 0.531 |

^a,b^Antibiotic concentration: ^a^ampicillin (10 µg), ^b^trimethoprim-sulfamethoxazole (1.25/23.75 µg) ^1^Samples collected at Day 0, pre-intervention ^2^Samples collected at Day 0, post-intervention p-values from Fisher’s exact test

**Supplementary Table 3b: Prevalence of *E. coli* gentamicin and ciprofloxacin resistance in different biological samples from women and their infants**

|  | **Prevalence of *E. coli* gentamicin^a^ resistance** | | | | **Prevalence of *E. coli* ciprofloxacin^b^ resistance** | | | |
| --- | --- | --- | --- | --- | --- | --- | --- | --- |
|  | **AZM**  **n/N (%)** | **Placebo n/N (%)** | **PR (95% CI)** | **p-value** | **AZM**  **n/N (%)** | **Placebo n/N (%)** | **PR (95% CI)** | **p-value** |
| **Women - rectovaginal swab samples** | | | | | | | | |
| **Day 0^1^** | 7/244  (2.9) | 5/256  (2.0) | 1.47 (0.47-4.58) | 0.568 | 13/244  (5.3) | 11/256  (4.3) | 1.25 (0.57-2.73) | 0.677 |
| **Women - breast milk samples** | | | | | | | | |
| **Day 6** | 1/241 (0.4) | 0/253 | - | 0.488 | 2/241  (0.8) | 0/253 | - | 0.238 |
| **Day 28** | 0/222 | 1/243  (0.4) | - | 1.000 | 0/222 | 0/243 | - | - |
| **Month 4** | 0/189 | 0/207 | - | - | 0/189 | 0/207 | - | - |
| **Children - rectal swab samples** | | | | | | | | |
| **Day 0^2^** | 2/240 (0.8) | 0/252 | - | 0.237 | 1/240  (0.4) | 1/252  (0.4) | 1.05 (0.07-16.76) | 1.000 |
| **Day 6** | 12/238  (5.0) | 11/250 (4.4) | 1.15 (0.57-2.73) | 0.832 | 23/238  (9.7) | 27/250 (10.8) | 0.90 (0.53-1.52) | 0.766 |
| **Day 28** | 12/220  (5.5) | 19/240  (7.9) | 0.69 (0.34-1.39) | 0.353 | 33/220  (15.0) | 35/240  (14.6) | 1.02 (0.66-1.60) | 1.000 |
| **Month 4** | 17/188  (9.0) | 12/206  (5.8) | 1.55 (0.76-3.16) | 0.250 | 45/188  (23.9) | 53/206  (25.7) | 0.93(0.66-1.31) | 0.727 |

^a,b^Antibiotic concentration: ^a^gentamicin (10 µg), ^b^ciprofloxacin (5 µg) ^1^Samples collected at Day 0, pre-intervention ^2^Samples collected at Day 0, post-intervention p-values from Fisher’s exact test

**Supplementary Table 3c: Prevalence of *E. coli* cefoxitin resistance and ESBL carriage in different biological samples from women and their infants**

|  | **Prevalence of *E. coli* cefoxicitin^a^ resistance** | | | | **Prevalence of *E. coli* ESBL carriage** | | | |
| --- | --- | --- | --- | --- | --- | --- | --- | --- |
|  | **AZM**  **n/N (%)** | **Placebo n/N (%)** | **PR (95% CI)** | **p-value** | **AZM**  **n/N (%)** | **Placebo**  **n/N (%)** | **PR (95% CI)** | **p-value** |
| **Women - rectovaginal swab samples** | | | | | | | | |
| **Day 0^1^** | 0/244 | 1/256 (0.4) | - | 1.000 | **5/244 (2.0)** | **0/256** | **-** | **0.027** |
| **Children - rectal swab samples** | | | | | | | | |
| **Day 0^2^** | 2/240 (0.8) | 0/252 | - | 0.237 | 0/240 | 0/252 |  |  |
| **Day 6** | 1/238 (0.4) | 0/250 | - | 0.487 | 7/238 (2.9) | 11/250  (4.4) | 0.67 (0.26-1.70) | 0.475 |
| **Day 28** | **0/220** | **7/240**  **(2.9)** | **-** | **0.016** | 12/220  (5.5) | 16/240  (6.7) | 0.82 (0.40-1.69) | 0.697 |
| **Month 4** | 3/188 (1.6) | 2/206 (1.0) | 1.64 (0.28-9.73) | 0.673 | 17/188  (9.0) | 22/206  (10.7) | 0.85 (0.46-1.54) | 0.616 |

^a^Antibiotic concentration: ^a^cefoxitin (30 μg), Antibiotic concentrations for ESBL detection: cefotaxime and cefotaxime + amoxicillin clavulanic acid [CT/CTL: cefotaxime (0.25-16 μg/mL)/cefotaxime (0.016-1 μg/mL) + clavulanic acid (4 μg/mL)] and ceftazidime and ceftazidime + amoxicillin clavulanic acid [TZ/TZL: ceftazidime (0.5-32 μg/mL)/ceftazidime (0.064-4 μg/mL) + clavulanic acid (4 μg/mL) ^1^Samples collected at Day 0, pre-intervention ^2^Samples collected at Day 0, post-intervention p-values from Fisher’s exact test

**Supplementary Table 4a: Prevalence of *K. pneumoniae* carriage in different biological samples from women and their infants (stratified by country)**

|  | **The Gambia** | | | | **Burkina Faso** | | | |
| --- | --- | --- | --- | --- | --- | --- | --- | --- |
|  | **Prevalence of carriage** | | | | **Prevalence of carriage** | | | |
|  | **AZM**  **n/N (%)** | **Placebo n/N (%)** | **PR (95% CI)** | **p-value** | **AZM**  **n/N (%)** | **Placebo n/N (%)** | **PR (95% CI)** | **p-value** |
| **Women - rectovaginal swab samples** | | | | | | | | |
| **Day 0^1^** | 32/122 (26.2) | 33/128 (25.8) | 1.02 (0.67-1.55) | 1.000 | 35/122 (28.7) | 35/128  (27.3) | 1.05 (0.71-1.56) | 0.888 |
| **Women – nasopharyngeal swab samples** | | | | | | | | |
| **Day 0^1^** | 4/121  (3.3) | 8/126  (6.3) | 0.52 (0.16-1.68) | 0.377 | 0/122 | 2/128  (1.6) | - | 0.498 |
| **Day 6** | **7/118 (5.9)** | **1/124**  **(0.8)** | **7.36 (0.92-58.88)** | **0.032** | 1/122  (0.8) | 1/128  (0.8) | 1.0 (0.07-16.59) | 1.000 |
| **Women - breast milk samples** | | | | | | | | |
| **Day 6** | 9/120 (7.5) | 6/125  (4.8) | 1.56 (0.57-4.26) | 0.432 | 3/121 (2.5) | 6/128  (4.7) | 0.53 (0.14-2.07) | 0.501 |
| **Day 28** | 8/101 (7.9) | 5/117  (4.3) | 1.85 (0.63-5.49) | 0.391 | 4/121  (3.3) | 2/127  (1.6) | 2.10 (0.39-11.25) | 0.437 |
| **Month 4** | 3/69  (4.3) | 2/83  (2.4) | 1.80 (0.31-10.49) | 0.659 | 2/120 (1.7) | 1/124  (0.8) | 2.07 (0.19-22.49) | 0.617 |
| **Children - rectal swab samples** | | | | | | | | |
| **Day 0^2^** | 3/120 (2.5) | 5/127  (3.9) | 0.64 (0.16-2.60) | 0.723 | 4/120 (3.3) | 3/125  (2.4) | 1.39 (0.32-6.08) | 0.718 |
| **Day 6** | **47/119 (39.5)** | **31/122 (25.4)** | **1.55 (1.07-2.27)** | **0.027** | 71/119 (59.7) | 62/128 (48.4) | 1.23 (0.98-1.55) | 0.097 |
| **Day 28** | **49/101 (48.5)** | **35/114 (30.7)** | **1.58 (1.12-2.22)** | **0.008** | **69/119**  **(58.0)** | **44/126 (34.9)** | **1.66 (1.25-2.20)** | **<0.001** |
| **Month 4** | 15/69 (21.7) | 22/82 (26.8) | 0.81 (0.46-1.44) | 0.570 | 23/119 (19.3) | 25/124 (20.2) | 0.96 (0.58-1.59) | 1.000 |
| **Children - nasopharyngeal swab samples** | | | | | | | | |
| **Day 0^2^** | 1/120 (0.8) | 0/127 | - | 0.486 | 0/120 | 0/126 | - | - |
| **Day 6** | 12/118 (10.2) | 8/124  (6.5) | 1.58 (0.67-3.72) | 0.354 | 8/119  (6.7) | 9/128  (7.0) | 0.96 (0.38-2.40) | 1.000 |
| **Day 28** | 4/101  (4.0) | 5/114  (4.4) | 0.89 (0.25-3.24) | 1.000 | 0/119 | 0/126 | - | - |
| **Month 4** | 0/69 | 0/82 | - | - | 1/119 (0.8) | 0/124 | - | 0.492 |

^1^Samples collected at Day 0, pre-intervention ^2^Samples collected at Day 0, post-intervention p-values from Fisher’s exact test

**Supplementary Table 4b: Prevalence of *K. pneumoniae* azithromycin resistance in different biological samples from women and their infants (stratified by country)**

|  | **The Gambia** | | | | **Burkina Faso** | | | |
| --- | --- | --- | --- | --- | --- | --- | --- | --- |
|  | **Prevalence of azithromycin^a^ resistance** | | | | **Prevalence of azithromycin^a^ resistance** | | | |
|  | **AZM**  **n/N (%)** | **Placebo n/N (%)** | **PR (95% CI)** | **p-value** | **AZM**  **n/N (%)** | **Placebo n/N (%)** | **PR (95% CI)** | **p-value** |
| **Women - rectovaginal swab samples** | | | | | | | | |
| **Day 0^1^** | 0/122 | 2/128 (1.6) | - | 0.498 | 1/122 (0.8) | 2/128  (1.6) | 0.52 (0.05-5.71) | 1.000 |
| **Women – nasopharyngeal swab samples** | | | | | | | | |
| **Day 0^1^** | 0/121 | 1/126 (0.8) | - | 1.000 | 0/122 | 0/128 |  |  |
| **Day 6** | 0/118 | 0/124 | - | - | 0/122 | 0/128 |  |  |
| **Women - breast milk samples** | | | | | | | | |
| **Day 6** | 3/120  (2.5) | 0/125 | - | 0.116 | 0/121 | 1/128 (0.8) | - | 1.000 |
| **Day 28** | 1/101  (1.0) | 0/117 | - | 0.463 | 0/121 | 0/127 | - | - |
| **Month 4** | 0/67 | 0/77 | - | - | 0/120 | 0/124 | - | - |
| **Children - rectal swab samples** | | | | | | | | |
| **Day 0^2^** | 0/120 | 0/127 | - |  | 0/120 | 1/125 | - | 1.000 |
| **Day 6** | 7/119  (5.9) | 2/122 (1.6) | 3.59 (0.76-16.92) | 0.099 | 7/119 (5.9) | 4/128 (3.1) | 1.88 (0.57-6.27) | 0.363 |
| **Day 28** | 5/101  (5.0) | 2/114 (1.8) | 2.82 (0.56-14.23) | 0.257 | **11/119 (9.2)** | **3/126 (2.4)** | **3.88 (1.11-13.57)** | **0.027** |
| **Month 4** | 0 /69 | 1/82  (1.2) |  | 1.000 | 3/119 (2.5) | 3/124 (2.4) | 1.04 (0.21-5.06) | 1.000 |

^a^Antibiotic concentration: ^a^azithromycin (0.016-256µg/mL), ^1^Samples collected at Day 0, pre-intervention ^2^Samples collected at Day 0, post-intervention p-values from Fisher’s exact test

| **Prevalence of *K. pneumoniae* azithromycin resistance** | | | | | **Prevalence of *K. pneumoniae* trimethoprim-sulfamethoxazole resistance** | | | |
| --- | --- | --- | --- | --- | --- | --- | --- | --- |
|  | **AZM**  **n/N (%)** | **Placebo n/N (%)** | **PR (95% CI)** | **p-value** | **AZM**  **n/N (%)** | **Placebo**  **n/N (%)** | **PR (95% CI)** | **p-value** |
| **Day 0^2^** | 0/7 | 1/8  (12.5) | - | 1.000 | 1/7  (14.3) | 2/8  (25.0) | 0.57 (0.06-5.03) | 1.000 |
| **Day 6** | 13/118 (11.0) | 6/93  (6.5) | 1.71 (0.67 - 4.32) | 0.334 | 30/118  (25.4) | 33/93  (35.5) | 0.72 (0.47-1.08) | 0.131 |
| **Day 28** | 16/118 (13.6) | 5/79  (6.3) | 2.14 (0.82 – 5.61) | 0.157 | **50/118**  **(42.4)** | **21/79**  **(26.6)** | **1.59 (1.04-2.43)** | **0.034** |
| **Month 4** | 3/38  (7.9) | 4/47  (8.5) | 0.93 (0.22 – 3.89) | 1.000 | 10/38  (26.3) | 17/47  (36.2) | 0.73 (0.38-1.40) | 0.359 |
| **Prevalence of *K. pneumoniae* gentamicin resistance** | | | | | **Prevalence of *K. pneumoniae* ciprofloxacin resistance** | | | |
| **Day 0^2^** | 2/7  (28.6) | 1/8  (12.5) | 2.29 (0.26-20.13) | 0.569 | 1/7  (14.3) | 1/8  (12.5) | 1.14 (0.09-15.08) | 1.000 |
| **Day 6** | 13/118  (11.0) | 9/93  (9.7) | 1.14 (0.51-2.55) | 0.823 | 17/118  (14.4) | 11/93  (11.8) | 1.22 (0.60-2.47) | 0.684 |
| **Day 28** | 23/118  (19.5) | 12/79  (15.2) | 1.28 (0.68-2.43) | 0.569 | 34/118  (28.8) | 14/79  (17.7) | 1.63 (0.93-2.83) | 0.091 |
| **Month 4** | 4/38  (10.5) | 7/47  (14.9) | 0.71 (0.22-2.24) | 0.747 | 5/38  (13.2) | 12/47  (25.5) | 0.52 (0.20-1.33) | 0.183 |
| **Prevalence of *K. pneumoniae* cefoxitin resistance** | | | | | **Prevalence of *K. pneumoniae* ESBL carriage** | | | |
| **Day 0^2^** | 0/7 | 2/8  (25.0) | - | 0.467 | 0/7 | 0/8 | - | - |
| **Day 6** | 3/118  (2.5) | 0/93 | - | 0.257 | 10/118  (8.5) | 4/93  (4.3) | 1.97 (0.64-6.08) | 0.274 |
| **Day 28** | 2/118  (1.7) | 2/79  (2.5) | 0.67 (0.10-4.65) | 1.000 | 21/118  (17.8) | 8/79  (10.1) | 1.76 (0.82-3.77) | 0.155 |
| **Month 4** | 0/38 | 1/47  (2.1) | - | 1.000 | 2/38  (5.3) | 5/47  (10.6) | 0.49 (0.10-2.41) | 0.453 |

**Supplementary Table 5: Frequency of antibiotic resistance in *K. pneumoniae* from rectal swab (RS) samples from infants (*K. pneumoniae* positive samples as denominator)**

Antibiotic concentrations as shown in Table 2 and Supplementary Tables 3a, 3b and 3c ^2^Samples collected at Day 0, post-intervention p-values from Fisher’s exact test

**Supplementary Table 6a: Prevalence of *K. pneumoniae* trimethoprim-sulfamethoxazole resistance in different biological samples from women and their infants**

|  | **Prevalence of *K. pneumoniae* trimethoprim-sulfamethoxazole ^a^ resistance** | | | |
| --- | --- | --- | --- | --- |
|  | **AZM**  **n/N (%)** | **Placebo n/N (%)** | **PR (95% CI)** | **p-value** |
| **Women - rectovaginal swab samples** | | | | |
| **Day 0^1^** | 15/244  (6.1) | 21/256 (8.2) | 0.75 (0.40-1.42) | 0.393 |
| **Women - breast milk samples** | | | | |
| **Day 6** | 7/241  (2.9) | 4/253  (1.6) | 1.84 (0.54-6.20) | 0.372 |
| **Day 28** | 3/222  (1.4) | 2/243  (0.8) | 1.65 (0.28-9.78) | 0.673 |
| **Month 4** | 2/189  (1.1) | 2/207  (1.0) | 1.10 (0.16-7.70) | 1.000 |
| **Children - rectal swab samples** | | | | |
| **Day 0^2^** | 1/240  (0.4) | 2/252  (0.8) | 0.53 (0.05-5.75) | 1.000 |
| **Day 6** | 34/238 (14.3) | 35/250 (14.0) | 1.02 (0.66-1.58) | 1.000 |
| **Day 28** | **51/220 (23.2)** | **21/240 (8.8)** | **2.65 (1.65-4.26)** | **<0.001** |
| **Month 4** | 10/188  (5.3) | 17/206 (8.3) | 0.64 (0.30-1.37) | 0.319 |

^a^Antibiotic concentration: ^a^trimethoprim-sulfamethoxazole (1.25/23.75µg) ^1^Samples collected at Day 0, pre-intervention ^2^Samples collected at Day 0, post-intervention p-values from Fisher’s exact test

**Supplementary Table 6b: Prevalence of *K. pneumoniae* gentamicin and ciprofloxacin resistance in different biological samples from women and their infants**

|  | **Prevalence of *K. pneumoniae* gentamicin^a^ resistance** | | | | | **Prevalence of *K. pneumoniae* ciprofloxacin^b^ resistance** | | | |
| --- | --- | --- | --- | --- | --- | --- | --- | --- | --- |
|  | **AZM**  **n/N (%)** | **Placebo n/N (%)** | **PR (95% CI)** | **p-value** | | **AZM**  **n/N (%)** | **Placebo n/N (%)** | **PR (95% CI)** | **p-value** |
| **Women - rectovaginal swab samples** | | | | | | | | | |
| **Day 0^1^** | 5/244 (2.0) | 4/256  (1.6) | 1.31 (0.36-4.83) | 1.000 | | 3/244  (1.2) | 3/256  (1.2) | 1.05 (0.21-5.15) | 1.000 |
| **Women - breast milk samples** | | | | | | | | | |
| **Day 6** | 1/241 (0.4) | 2/253  (0.8) | 0.53 (0.05-5.75) | | 1.000 | 0/241 | 1/253  (0.4) | - | 1.000 |
| **Day 28** | 3/222 (1.4) | 2/243  (0.8) | 1.65 (0.28-9.78) | | 0.673 | 2/222  (0.9) | 1/243  (0.4) | 2.20 (0.20-24.08) | 0.607 |
| **Month 4** | 1/189 (0.5) | 1/207  (0.5) | 1.10 (0.07-17.39) | | 1.000 | 0/189 | 0/207 | - | - |
| **Children - rectal swab samples** | | | | | | | | | |
| **Day 0^2^** | 2/240 (0.8) | 1/252  (0.4) | 2.1 (0.19-23.01) | 0.615 | | 1/240  (0.4) | 1/252  (0.4) | 1.05 (0.07-16.69) | 1.000 |
| **Day 6** | 14/238 (5.9) | 10/250  (4) | 1.47 (0.67-3.25) | 0.404 | | 20/238 (8.4) | 12/250 (4.8) | 1.75 (0.88-3.50) | 0.143 |
| **Day 28** | **23/220 (10.5)** | **12/240**  **(5.0)** | **2.09 (1.07-4.10)** | **0.034** | | **34/220 (15.5)** | **14/240 (5.8)** | **2.65 (1.46-4.80)** | **0.001** |
| **Month 4** | 4/188 (2.1) | 7/206  (3.4) | 0.63 (0.19-2.10) | 0.548 | | 5/188  (2.7) | 12/206 (5.8) | 0.46 (0.16-1.27) | 0.142 |

^a,b^Antibiotic concentration: ^a^gentamicin (10µg), ^b^ciprofloxacin (5µg) ^1^Samples collected at Day 0, pre-intervention ^2^Samples collected at Day 0, post-intervention p-values from Fisher’s exact test

|  | **AZM**  **n/N (%)** | **Placebo n/N (%)** | **PR (95% CI)** | **p-value** | **AZM**  **n/N (%)** | **Placebo n/N (%)** | **PR (95% CI)** | **p-value** |
| --- | --- | --- | --- | --- | --- | --- | --- | --- |
| **Women - rectovaginal swab samples** | | | | | | | | |
| **Day 0^1^** | **5/244**  **(2.0)** | **0/256** | **-** | **0.027** | 0/244 | 1/256 (0.4) | - | 1.000 |
| **Children - rectal swab samples** | | | | | | | | |
| **Day 0^2^** | 0/240 | 2/252 (0.8) | - | 0.499 | 0/240 | 0/252 | - | - |
| **Day 6** | 3/238  (1.3) | 0/250 | - | 0.115 | 10/238  (4.2) | 4/250 (1.6) | 2.63 (0.83-8.26) | 0.106 |
| **Day 28** | 2/220  (0.9) | 2/240 (0.8) | 0.09 (0.15-7.68) | 1.000 | **21/220**  **(9.5)** | **8/240 (3.3)** | **2.86 (1.30-6.33)** | **0.007** |
| **Month 4** | 0/188 | 1/206 (0.5) | - | 1.000 | 2/188  (1.1) | 5/206 (2.4) | 0.44 (0.09-2.23) | 0.452 |

**Supplementary Table 6c: Prevalence of *K. pneumoniae* cefoxitin resistance and ESBL carriage in different biological samples from women and their infants**

^a^Antibiotic concentration: ^a^cefoxitin (30 μg), Antibiotic concentrations for ESBL detection: cefotaxime and cefotaxime + amoxicillin clavulanic acid [CT/CTL: cefotaxime (0.25-16 μg/mL)/cefotaxime (0.016-1 μg/mL) + clavulanic acid (4 μg/mL)] and ceftazidime and ceftazidime + amoxicillin clavulanic acid [TZ/TZL: ceftazidime (0.5-32 μg/mL)/ceftazidime (0.064-4 μg/mL) + clavulanic acid (4 μg/mL) ^1^Samples collected at Day 0, pre-intervention ^2^Samples collected at Day 0, post-intervention p-values from Fisher’s exact test

Supplementary figure 1: Study sites in the Gambia (left) and Burkina Faso (right).

Supplementary Figure 2: Study sample types and bacteria isolated.

Supplementary Figure 3: RS *E.coli* and *K. pneumoniae* antibiotic resistance at days 6 and 28.

RS = Rectal swab

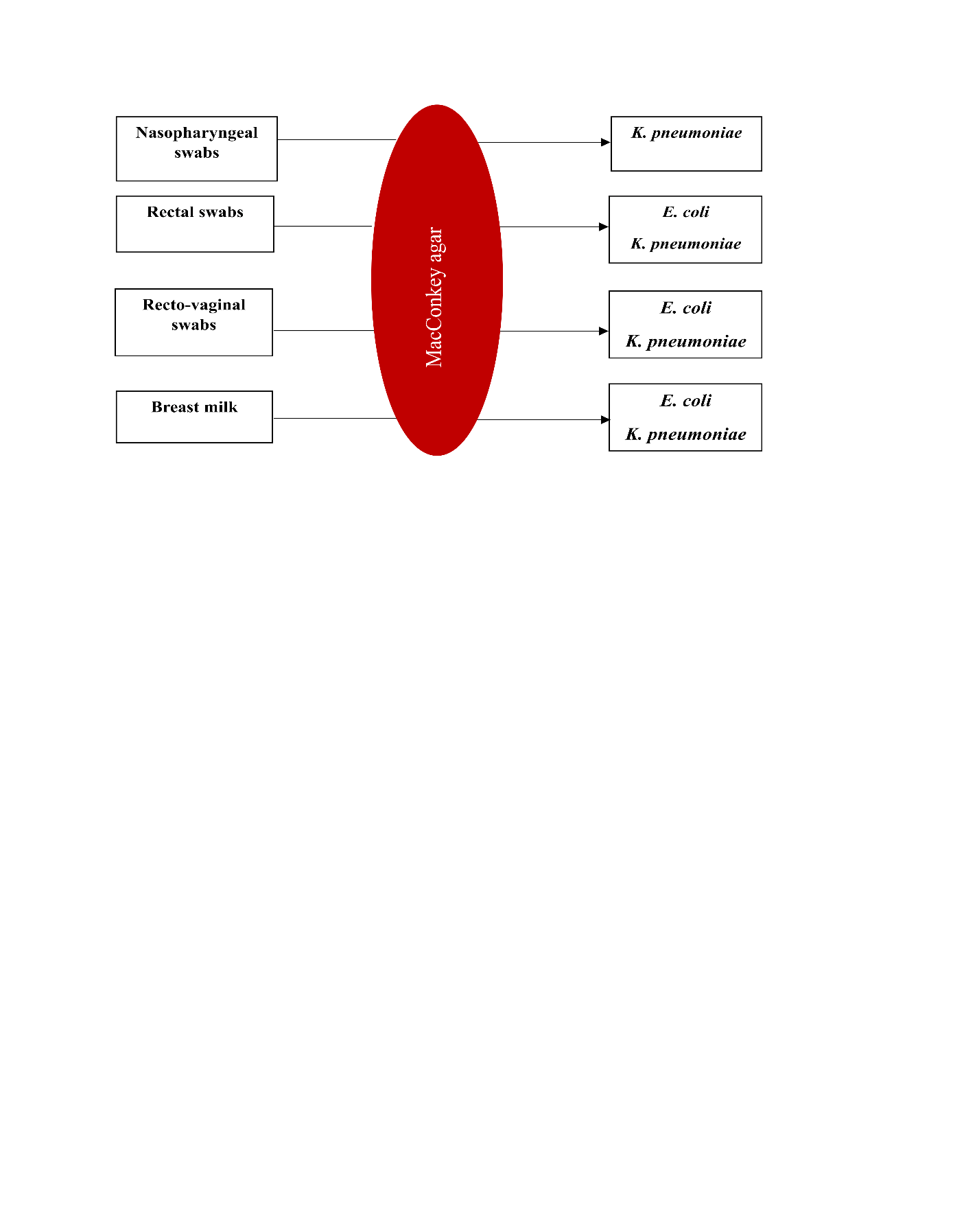


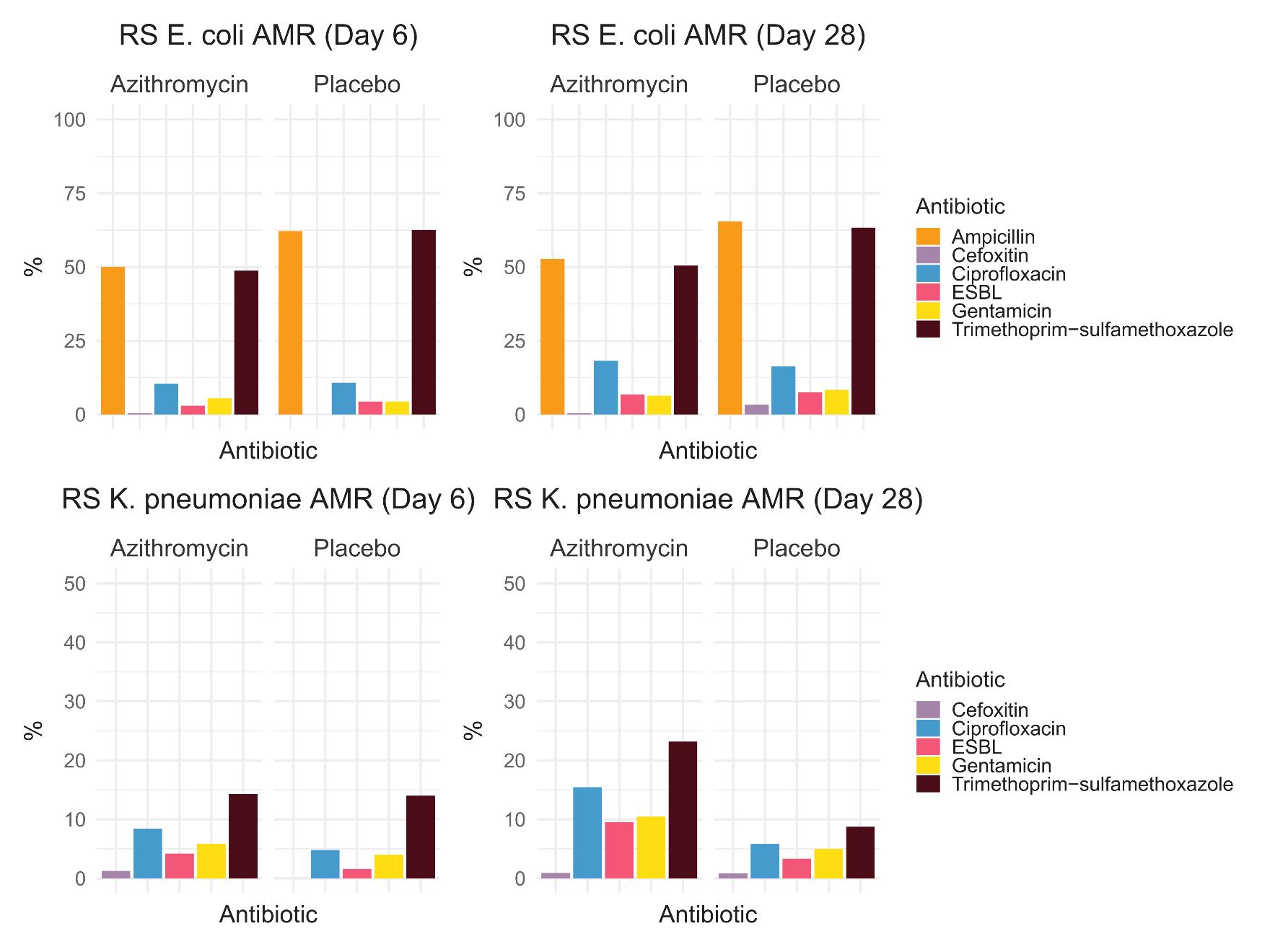

Supplement: ciae280_Supplementary_Data [file ciae280_supplementary_data.docx]
